# The effects of network topology, climate variability and shocks on the evolution and resilience of a food trade network

**DOI:** 10.1371/journal.pone.0213378

**Published:** 2019-03-26

**Authors:** Alexander G. Dolfing, Jasper R. F. W. Leuven, Brian J. Dermody

**Affiliations:** 1 Copernicus Institute of Sustainable Development, Faculty of Geosciences, Utrecht University, Utrecht, the Netherlands; 2 Natuur & Milieu, organization for the protection of nature and the environment, Utrecht, the Netherlands; 3 Department of Physical Geography, Faculty of Geosciences, Utrecht University, Utrecht, the Netherlands; Uniiversity of Padova, ITALY

## Abstract

Future climate change will impose increased variability on food production and food trading networks. However, the effect of climate variability and sudden shocks on resource availability through trade and its subsequent effect on population growth is largely unknown. Here we study the effect of resource variability and network topology on access to resources and population growth, using a model of population growth limited by resource availability in a trading network. Resources are redistributed in the network based on supply and the distance between nodes (i.e. cities or countries). Resources at nodes vary over time with wave parameters that mimic changes in biomass production arising from known climate variability. Random perturbations to resources are applied to study resilience of individual nodes and the system as a whole. The model demonstrates that redistribution of resources increases the maximum population that can be supported (carrying capacity) by the network. Fluctuations in carrying capacity depend on the amplitude and frequency of resource variability: fluctuations become larger for increasing amplitude and decreasing frequency. Our study shows that topology is the key factor determining the carrying capacity of a node. In larger networks the carrying capacity increases and the distribution of resources in the network becomes more equal. The most central nodes achieve a higher carrying capacity than nodes with a lower centrality. Moreover, central nodes are less susceptible to long-term resource variability and shocks. These insights can be used to understand how worldwide equitable access to resources can be maintained under increasing climate variability.

## Introduction

Climate impacts all life on Earth, because variability in temperature and water availability affect primary productivity and thus food availability at all trophic levels. It is known that organisms compete for food resources in variable environments [1]. However, little is known about the fundamental dynamics underlying how humans access food resources in variable environments. Humans, like other organisms, are impacted by changes in food availability arising from climate variability [2]. Since the beginning of civilization, humans have developed methods to provide buffers against climate variability such as the use of irrigation, food stocks and resource redistribution via trade [3; 4]. Like other social organisms, such as ants, these strategies are associated with a fundamental reorganization of our societal structure [5].

For example, resource redistribution via trade is intimately connected with the growth of cities. In fact, trade and cities coevolved because the trade networks that radiate from cities enabled human populations to overshoot their local carrying capacities, which opened up the possibility to agglomerate in one location [6]. The Earth is currently inhabited by 7.5 billion people, more than half of whom reside in cities and are therefore dependent on trade to secure access to most of their resources. These cities competitively interact for a range of resources, such as food, products and services to sustain growth. The rapid growth of the human population presents a challenges to supply of sufficient food to all who need it. It is estimated that global population will reach 9 billion by 2050 with 65% of those people residing in cities [7].

Food trade also provides a buffer against local variability in food resources because regions can import when they have a deficit and export when they have a surplus. The redistribution of resources from regions with surplus to regions with deficit effectively increases the carrying capacity of a trading network [8]. The reason is that under multi-year to decadal climate variability, the carrying capacity of a non-trading region is limited by the food availability in the periods with the lowest yield. However, when regions with heterogeneous climate are connected via a trade network, the carrying capacity approaches the average carrying capacity of all trading nodes. Trade can thus increase the carrying capacity of a trade network without any increase in production [8]. However, the interdependency of food supply via trade means that changes in one part of a trade network may have implications for food supply in another part of the network. This interdependence of regions for food resources has been observed during recent food price crises of 2007-2008 and 2010-2011 [9]. The price surges were induced by a combination of extreme weather and environmental events (e.g. wildfires, droughts) which led to a reduction of food on global markets leading to an increase in food prices for trading nations [10]. A study of virtual water trade in the ancient world demonstrated that trade can save resources on average [8], but other studies show that increased reliance on trade increases vulnerability to food shortages in the real world trading network [9; 11]. The challenge of food supply will be complicated even further by anthropogenic climate change, which alters the frequency and amplitude of climate variables such as rainfall and temperature patterns [12]. These effects of climate change will likely be most acute in poorer nations where an increase in food prices may lead to hunger [13].

Besides long-term effects on food supply, sudden shortages of food may occur when a harvest fails in a certain region due to extreme weather events, pest outbreaks etc. This can result in sudden declines in export volumes of food (i.e. shocks) from these regions when they decide to stop exporting. Previous studies on the effect of such shocks on food availability in the real-world food trade networks, using a combination of empirical and modelling approaches, provide important insights about the effects and propagation of these shocks [e.g.] [9; 11; 13; 14; 15; 16; 17; 18]. They found that import dependent and water-scarce countries are most vulnerable to shocks [16; 17], and that especially developing countries experience the highest losses in import volume due to these sudden decreases in export volume in the network [18]. Moreover, these shocks can alter the network structure, where trading entities increase their number of trading partners after they have been affected by a shock [15]. Countries can increase their resilience against shocks by increasing the number and diversity of their trading partners and increasing food buffer stocks [11; 9].

In this paper we focus on understanding the role that climate variability, network topology and shocks have in terms of access to resources in a trading network. We present a simple model that allows us to isolate the effect of these factors. The dynamics identified in our study are intended to provide insights that are generalisable to food trade networks.

## Materials and methods

**Fig 1:**
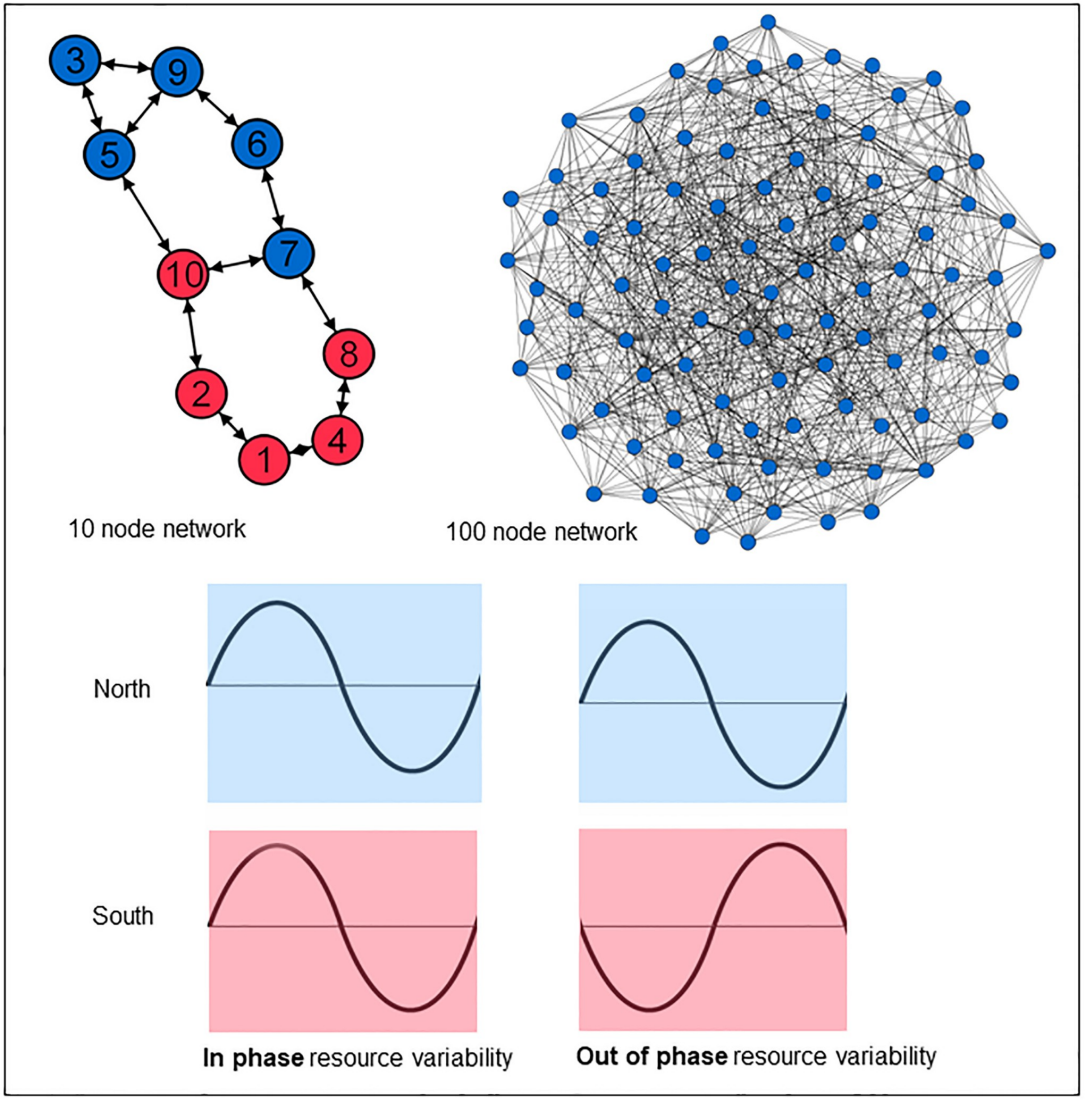
Conceptual overview of the networks and resource variability scenarios. The networks on which our analysis was carried out and a visualisation of the resource variability are shown. The networks are small-world and undirected, compromising 10 and 100 nodes. Resource availability at nodes varies over time in the form of a sine wave. With in phase variability, all nodes experience the same variability. In out of phase the *north* group (blue) experiences a sine wave 180 degrees out of phase with the *south* group (red), as illustrated by the colors in the 10 node network.

To summarize our methodology; we generated a food distribution system of 10 and 100 nodes with a small-world network configuration. Population growth was simulated in these networks as a function of local resources at the node and competition for resources in the rest of the network. Resources varied in the network with varying amplitude and periodicity to reflect multiannual to decadal oscillation in the climate system where frequency occurs in phase or out of phase. Fig 1 and S1 Fig illustrate the experimental setup.

### Network

All simulations were carried out on networks which were generated using the Watts-Strogatz model for producing networks with small-world characteristics [19]. Small-world networks were used as they show a high efficiency in distributing resources with low costs. This is explained by the topology of the network, in which most nodes trade locally but a few trade globally, allowing goods to be traded from far away with relatively few exchanges. Markets, such as the food market, show these same characteristics of local exchange and global reach [20].

Our analysis was carried out on small-world networks in sizes of 10 and 100 nodes (Fig 1). The links between nodes are fixed to measure the impact of network topology on the population of a node (NEP) in response to climate variability and shocks. The distance between the nodes was expressed as the number of edges on the shortest pathway between the nodes. The layout of the network, which is relevant when applying spatially autocorrelated climate variability, was produced by MATLAB through the function digraph, which creates a weighted directed graph object on the method developed by Fruchterman et al. (1991) [21]. A sensitivity analysis on the effect of network layout produced by the Watts-Strogatz model was conducted by running the model with 100 randomly generated networks of 10 and 100 nodes (S2 and S3 Figs). The model is generalisable and may represent countries, cities or other spatially constrained trading entities.

### Population growth and resource trade

The model runs for 1000 iterations, so an equilibrium state is achieved in every scenario. Population growth is calculated at each node within the network based on their carrying capacity. The starting population at every node is 1250 people to reduce run-up time. The carrying capacity of a node [*K*(*t*) in number of people] is based on local production of resources and import through trade. Local resource production (*R*(*t*)) is variable over time with wave parameters to reflect climate variability.

The equation for local production at a node is as follows:

$$\begin{array}{c} R(t)=\mu +x(t) \end{array}$$ (1)

where *R*(*t*) are the resources for a given node in year t, *μ* is the mean resource production, set at 1 million kg of food resources, which is sufficient to support 5000 people with a consumption of 200 kg per year per person. *x* is the deviation from the mean (*μ*), based on the year and the variability scenario.

In each node, 50% of the food is available for export while the other half is consumed locally. This reflects a system with resource specialization in which nodes have a certain demand for local and remote resources. Resources that are not consumed locally, a situation that occurs when population is low, are also available to other nodes in the network. Resources are redistributed among nodes using a trade equation (Eq 2) which is based on (a) the surplus and (b) the distance to the available resources, analogous to the gravity equation of trade [22]. Usually, the gravity model uses both demand and supply to estimate the flow of goods between two trading entities [22]. Here, we focus on resource availability in determining growth, rather than demand-driven dynamics, because that would imply that importing nodes have agency over exporting nodes. This may be the case, but we wish to solely focus on resource availability in determining carrying capacity. The flow of goods between nodes is represented by the following equation [22]:

$$\begin{array}{c} F_{ij}=\frac{M_{i}}{r_{ij}^{2}}\cdot \frac{\sum F}{\sum M} \end{array}$$ (2)

in which *F*_*ij*_ is the trade flow between two nodes, from node *i* to node *j*. *M*_*i*_ is the availability of resources for export (in kg) in node *i* and *r*_*ij*_ is the distance in number of edges between these nodes. The fraction $\frac{M_{i}}{r_{ij}^{2}}$ is multiplied by the total of flow between all nodes in the network (∑*F*) divided by the total availability of resources in the network, which is equal to 50% of total production reserved for export plus locally unconsumed resources (∑*M*). Flow *F*_*ij*_ will thus become larger if the availability is high and the distance short. Equally, as demand increases, the distance over which resources are imported increases. Through this equation, each node receives resources based on the availability in the network and the distance to nodes with a surplus.

Population growth at each node is calculated based on access to resources (carrying capacity), which is a sum of 50% of local resources and total import [23]:

$$\begin{array}{c} P\left(t\right)=\frac{K\left(t\right)P\left(t-1\right)e^{\alpha t}}{K\left(t\right)+P\left(t-1\right)(e^{\alpha t}-1)} \end{array}$$ (3)

in which *P*(*t*) is population at time step *t*, *α* is the yearly growth rate, and *K*(*t*) is the carrying capacity of the node. The yearly growth rate (*α*) was set to a constant of 1.2%, which is the estimated average global population growth rate in 2015 [24]. *K*(*t*) is the sum of local resources and imported resources per year.

We report the effect of network topology and resource variability on the growth and variation of the population in the nodes and networks through several variables. For network topology, the network size (number of nodes) and the closeness centrality of the nodes (which is a measure of the centrality of a node in the network) were used. Given that closeness centrality is smaller for larger networks because the sum of the paths becomes larger [25], the closeness centrality was normalized by dividing by the maximum value, such that values are always between 0 and 1. This allows for a comparison between networks of different sizes. The effects of resource variability were measured under different scenario’s, which are explained in the section below.

Two variables were used to report the size and variation in population. For population size in a node the Node Equilibrium Population (NEP) was used. This is the population of a node once the simulation has reached equilibrium. The population has reached equilibrium when the initial growth phase is over, and population has reached carrying capacity and thus numbers increase and decrease due to the variability in food availability. The average NEP of the nodes in a network is the Global Equilibrium Population (GEP). Both NEP and GEP are reported as an average over time after equilibrium or as the coefficient of variation, which reflects the variability of NEP and GEP over time after equilibrium and is defined as the standard deviation divided by the mean of NEP or GEP. This is different from the carrying capacity of the nodes, which reflects the maximum potential population that can be sustained at a given time by the availability of resources but may not be reached where the rate of change in resource variability exceeds the population growth rate.

### Climate variability scenarios

Resource availability at nodes varies over time in the form of a sine wave (see Fig 1 and Table 1). The amplitude is either 5% or 50% of the baseline resources. These were chosen to reflect respectively a low and high amplitude of variation. The periods (frequency) are 6 years (high frequency) or 70 years (low frequency), corresponding with periodicities of prominent climactic phenomena of El Niño-Southern Oscillation (ENSO) and the Atlantic Multidecadal Oscillation (AMO) respectively [26; 27]. To analyze the effect of resource variability on carrying capacity, a baseline scenario was used without resource variability. Subsequently, scenarios of resource variability were applied to the network as in phase or out of phase.

**Table 1:** The four different modes of variability. They are sine wave functions varying over a base of 1 million kg food resources per year. The amplitude in a mode can either be 5% or 50% and the period of the sine wave can either be 6 years (high frequency) or 70 years (low frequency).

Variability mode	abbreviation	Relative amplitude (%)	Period (years)
Low amplitude, high frequency	LAHF	5	6
low amplitude, low frequency	LALF	5	70
high amplitude, high frequency	HAHF	50	6
high amplitude, low frequency	HALF	50	70

With *in phase variability* all nodes in the network experience increases and decreases in resource production simultaneously. This is for example observed in countries on the western coast of South America that generally experience in phase signal of ENSO variability with all countries becoming anomalously wet or dry for under El Niño or La Niña conditions respectively [28].

To examine the role of *out of phase variability*, the network was divided into North and South clusters based on the calculated lay-out (see subsection Networks). The 50% with the highest x-coordinates were placed in the “North” cluster and the nodes with the lowest x-coordinates in the “South” cluster. Subsequently, the southern group is ascribed resource variability that is 180 degrees out of phase with the northern group. A phase difference of 180 degrees implies that maxima and minima occur simultaneously in opposite regions (see Fig 1 for an illustration). This scenario corresponds with actual climate variability which often results in opposite effects in different regions, such as dry circumstances in the Mediterranean and wetter than usual circumstances in northern Europe during a positive Northern Atlantic Oscillation (NAO) index [29]. Another example of the impact of out of phase climate variability on food production is the Trans-Pacific ENSO teleconnection between major crop producing regions in the America’s and those in Northern China or Australia. During an El Niño, maize and soybean growing conditions in the US and southeast South America are favourable, while the conditions are poor in northern China, the Cerrado in Brazil and southern Mexico [30].

Besides the gradual change in resource production due to climate variability, sudden changes in resource availability, shocks, were also studied. In this scenario, a shock was applied to one node, which suddenly stopped exporting food. However, this node could still import food from other nodes. The shock was applied to a hub node, a peripheral node and a node with median closeness centrality. This was done by calculating closeness centrality measures for all nodes in the network and selecting those nodes with the highest, lowest and median closeness centrality values respectively. With the incidence of a shock, resource redistribution was reduced by 1% in the 100 node network and 10% in the 10 node network. The shock node no longer exported the reserved 50% of its resources but used all resources for local consumption, reflecting the protectionist measure of voluntary export restraints taken in the real-world trade network [31].

## Results

### The effect of resource variability on NEP and GEP

**Fig 2:**
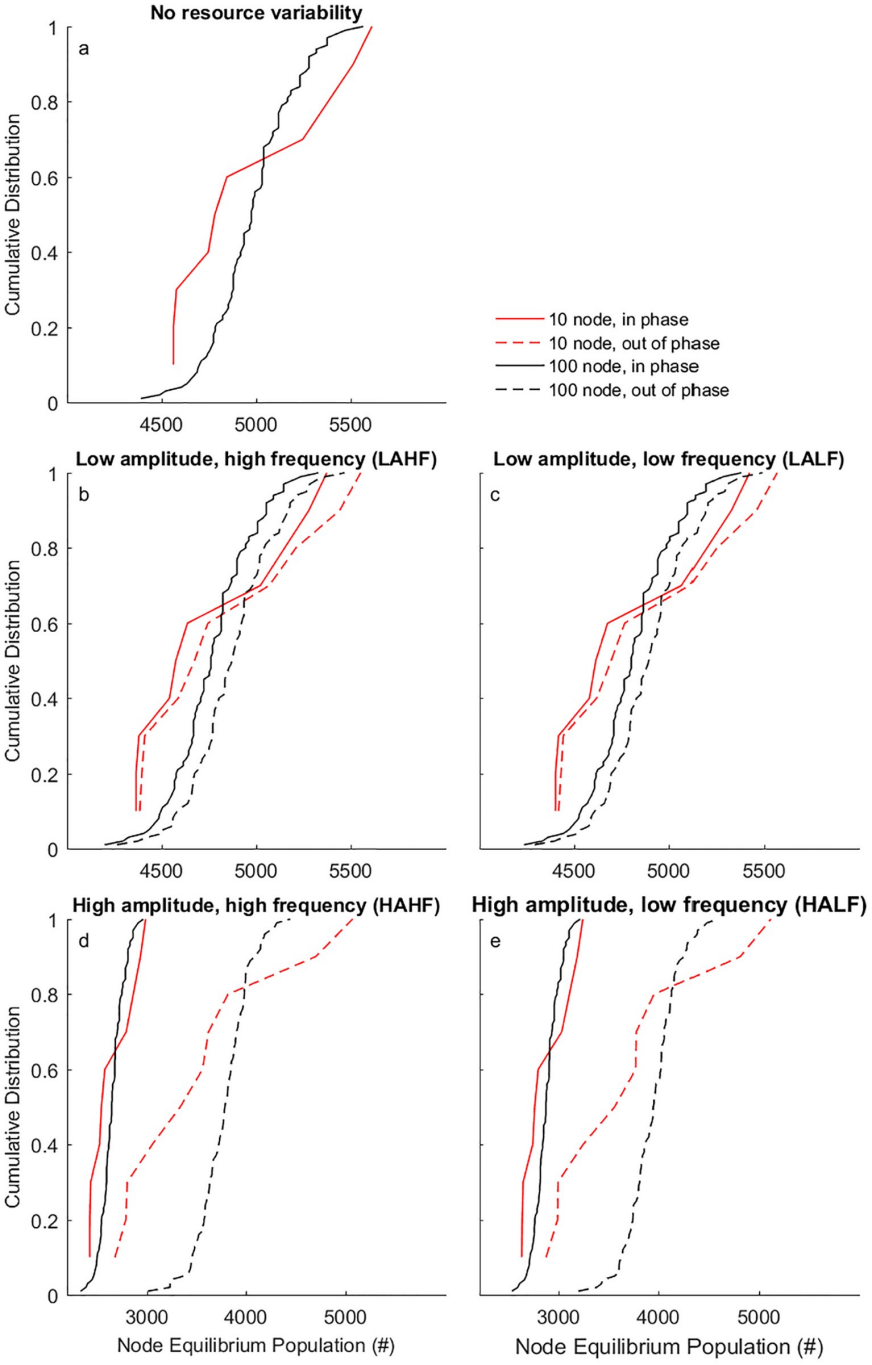
The cumulative distribution of NEP in the 10 and 100 node networks under the variability scenarios. (A) Cumulative distribution of NEP in the 10 node and 100 node networks. The cumulative fraction (y-axis) shows the proportion of nodes with a value less than or equal to the associated X value. (B,C,D,E) Effect of resource variability on NEP distributions within the networks. Solid lines indicate in phase resource variability. Dashed lines indicate 180 degrees out of phase resource variability.

The model results show that Node Equilibrium Population (NEP) and Global Equilibrium Population (GEP) depend on amplitude and frequency of resource production: in phase variability decreases the NEP for all nodes compared to the baseline scenario (Fig 2). In phase high amplitude (HA) variability results in lower population than in phase low amplitude (LA) variability. In the 100 node network, the low amplitude and high frequency (LAHF) variability causes a 4.3% decrease in GEP compared with the baseline, while low amplitude and low frequency (LALF) variability causes a 3.5% decrease in GEP (Table 2). High amplitude and high frequency (HAHF) variability results in a 46.7% decrease in GEP compared to the baseline, while high amplitude and low frequency (HALF) variability results in a 42.3% decrease in GEP. Thus, lower frequencies and lower amplitude variability result in higher GEP with in phase variability compared with high amplitude and high frequency variability. The increased GEP with lower frequencies of variability is caused by the growth rate of population: with lower frequency the population has more time to grow toward the increased carrying capacity. Still, the rate of change of the carrying capacity is sufficiently fast not to be tracked by population growth (1.2% per annum). Therefore, GEP is always lower than the maximum carrying capacity in a system with (in phase) variability, also with a low frequency of variability.

**Table 2:** The Global Equilibrium Population (GEP, the average population of the nodes in the networks after equilibrium) of the 10 and 100 node networks under the different resource variability scenarios: Low amplitude high frequency (LAHF), low amplitude low frequency (LALF), high amplitude high frequency (HAHF) and high amplitude low frequency (HALF). GEP is higher under out of phase variability and within the variability scenarios higher under lower amplitude and lower frequency of variability.

Resource variability scenario	10 node network	100 node network
No variability	4984	4984
LAHF in phase	4769	4769
LALF in phase	4812	4812
HAHF in phase	2653	2653
HALF in phase	2875	2874
LAHF out of phase	4848	4876
LALF out of phase	4877	4901
HAHF out of phase	3544	3780
HALF out of phase	3708	3941

In comparison to in phase, with out of phase variability NEP and GEP are increased in all four scenarios but are still lower than the baseline scenario (Fig 2, dashed lines). Interestingly, out of phase variability leads to larger differences in NEP among nodes, as can be seen from the shape of the cumulative distribution curves. This effect is especially pronounced under high amplitude variability. The increase in GEP under out of phase variability compared with in phase variability arises because resources can be redistributed from parts of the network with a surplus to parts of the network with a deficit. The larger inequality among nodes in NEP in out of phase variability arises because more resources are redistributed in out of phase and high amplitude variability scenarios. Compared with in phase variability, this means that the centrality of a node plays a more important role in determining access to resources in out of phase variability. As a result, the heterogeneity in node centrality give rise to greater inequality in NEP under out of phase variability.

Resource variability also affects the variation of GEP over time. Higher amplitudes and lower frequencies result in more variation of GEP over time (Table 3). Under in phase variability, the HALF scenario causes the highest variability in GEP whereas under LAHF, variation in GEP was 99% lower for all networks than under HALF. This difference is in part caused by the amplitude: with a higher amplitude the variation in resources over time is higher, resulting in a higher variation in population. However, frequency is the primary cause of variation in GEP. With high frequency, the population growth cannot track changes in resource availability and thus variability in GEP is lower, even when amplitude is high. When frequency is low, population has more time to grow and subsequently declines more when resource availability deceases. Out of phase resource variability results in a lower variation in population over time than in phase variability, because when one part of the network is low resources, another part is high in resources. Trade then results in a lower variation in GEP.

**Table 3:** The coefficient of variation of the Global Equilibrium Population (GEP, the average population of the nodes in the networks after equilibrium) over time in the different networks and the climate variability scenarios: Low amplitude high frequency (LAHF), low amplitude low frequency (LALF), high amplitude high frequency (HAHF) and high amplitude low frequency (HALF). GEP is more stable over time in out of phase scenario’s and within the variability scenarios more stable in higher frequency and lower amplitude of variability.

Resource variability scenario	10 node network	100 node network
LAHF in phase	0.0013	0.0013
LALF in phase	0.0107	0.0107
HAHF in phase	0.0111	0.0111
HALF in phase	0.1038	0.1037
LAHF out of phase	0.0009	0.0007
LALF out of phase	0.0073	0.0059
HAHF out of phase	0.0074	0.0061
HALF out of phase	0.0688	0.0561

### The effect of network topology on NEP and GEP

Network size does not affect GEP under in phase variability. However, with out of phase variability, GEP increases with increasing network size (Table 2). This effect of network size on GEP increases with increasing amplitude of variability. For example, the GEP of the 100 node network HALF variability is 6.3% larger than in the 10 node network.

Besides the effect on GEP, a larger network size reduces the difference in NEP between the most populated and least populated nodes. When the amplitude of resource variability is low (both in and out of phase), network size has little effect on population inequality. However, as resource variability increases in amplitude, smaller networks exhibit greater inequality in NEP compared with larger networks, shown in a less steep cumulative distribution curve (Fig 2D and 2E). The larger network results in more equality in NEP and higher GEP, because a denser network allows for better supply and demand interaction resulting from more nodes that can trade with each other. An important factor impacting the efficiency of resource redistribution is the spatial autocorrelation of variability. The spatial autocorrelation of variability is high, to reflect spatial autocorrelation of climate variability. As a result, nodes have the same variability as many of their neighbouring nodes, increasing the distance over which food needs to be imported. However, as network size increases further there is a decrease in variation of the population within the network. This is owing to the small-world nature of the network where maximal path-lengths remain low as network size increases. Thus, in small-world networks increasing network size will lower variation in carrying capacities caused by resource variability. The same mechanism explains why larger networks also result in a lower variation of GEP over time (Table 3). For example, under the out of phase HALF scenario, the coefficient of variation of GEP of the 100 node network is 18% lower than in the 10 node network. Larger small-world networks are thus more efficient at distributing resources among nodes.

**Fig 3:**
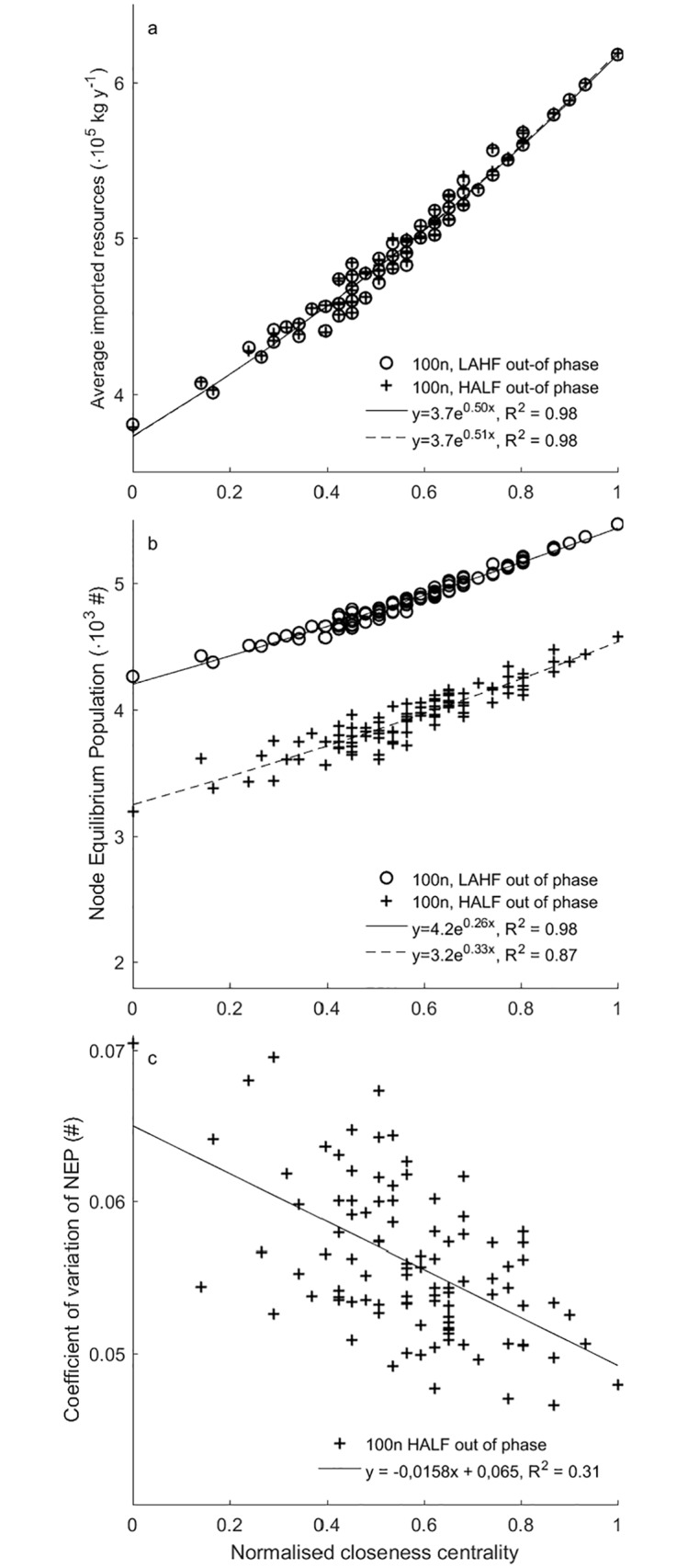
The effect of closeness centrality on Node Equilibrium Population. (A) Average resource import and (B) Node Equilibrium Population (NEP) as a function of the closeness centrality of the 100 node network under two out of phase variability scenarios: low amplitude high frequency (LAHF) and high amplitude low frequency (HALF). (C) The coefficient of variation of NEP as a function of closeness centrality for the high amplitude low frequency scenario (HALF).

Nodes with a more central position import more resources per year and can therefore sustain a larger NEP (Fig 3A and 3B). If centrality is high, the node has a higher trade flow resulting from the trade equation (Eq 2), and thus has access to more resources. Moreover, central nodes have a smaller variation in NEP over time (Fig 3C). This is owing to the low average distance to other nodes, which improves their access to resources in the network. When resource availability decreases in the network, more central nodes can access resources from more nodes and from nodes further away in the network, making them more resilient.

### Transmission of shocks

**Fig 4:**
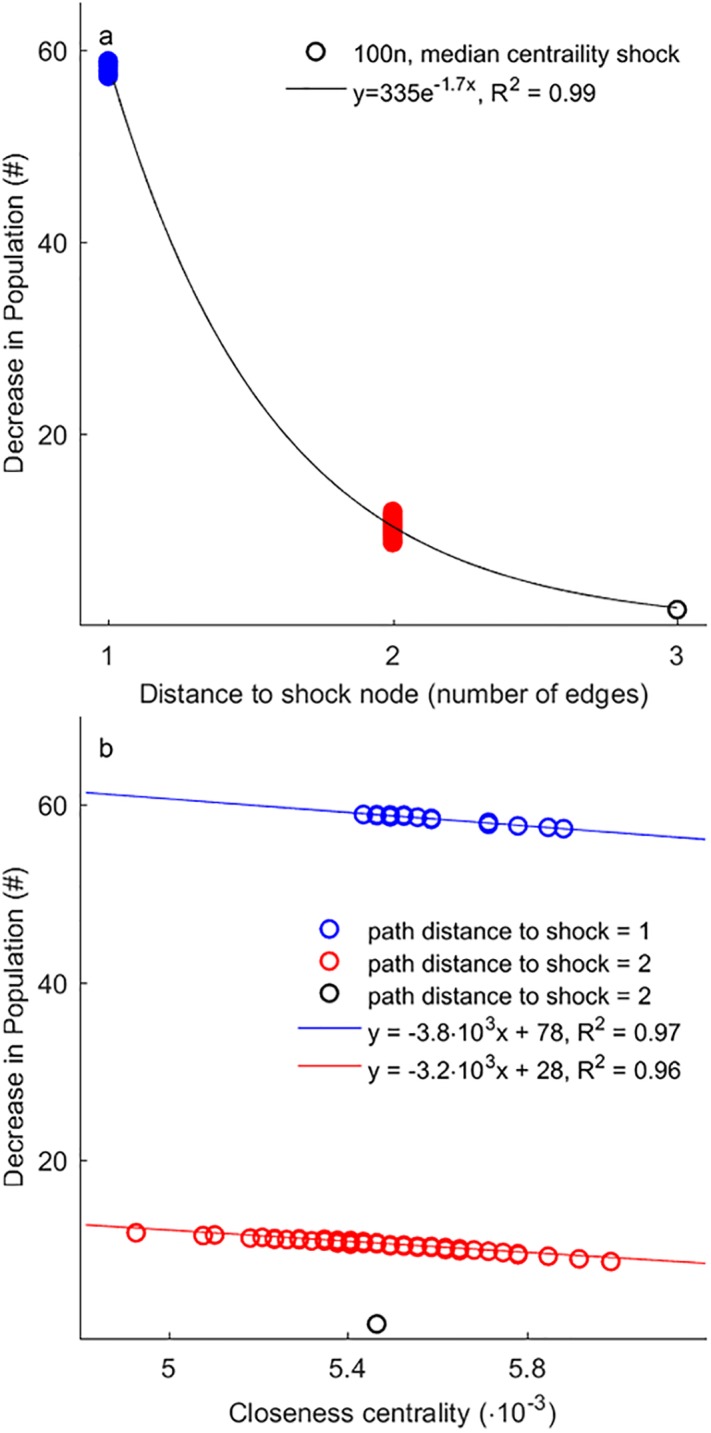
The effect of a shock on Node Equilibrium Population. The decrease in NEP after a shock as a function of (A) the distance to the shock node and (B) the closeness centrality.

Distance determines the sensitivity of a node to a shock. Nodes that are closer to the shock suffer larger population losses (Fig 4A). Nodes preferentially trade with close neighbours, because shorter distances reduce the cost of trade (Eq 2). Nodes therefore become dependent on an influx of resources from their neighbours. When this influx suddenly stops, as with a shock, the carrying capacity of the node is reduced, resulting in a loss of population.

The closeness centrality of a node also influences the population loss after a shock, as it is also a measure of distance. Central nodes are more protected against shocks than peripheral nodes (Fig 4B). A higher centrality results in favourable import conditions because it means that the average distance to the other nodes is relatively short, and therefore the node has easier access to resources in the network compared with a node that is in a more peripheral location in the network. Because there is still a high availability of resources in the network during a shock from all the other nodes, this results in less population loss for more central nodes after a shock. The distance to the shock and the distance to all other nodes in the network (closeness centrality) thus determine population loss after a shock. If two nodes are both directly connected to the shock node, they both suffer large damage. However, the node with the higher closeness centrality suffers less damage because it can more easily import resources from other nodes in the network, as it is relatively closer to these nodes.

The distance to a node and centrality are both a measure of dependency. Nodes depend more on trade from their close neighbours and less central nodes are more dependent on a single or a few nodes because their distance to most other nodes is long. These results show that a higher dependency of a node on a shock node increases the nodes population loss after a shock.

The position of the shock node influences the impact on GEP. Table 4 shows that the loss in GEP in the other nodes in the network is larger when the shock is applied to a node with a higher centrality. This can also be explained by the distance to a shock. The average distance from all other nodes to a central node is lower, therefore they depend more on the trade with the central node. Therefore, when a shock is applied to a central node, more nodes are impacted.

**Table 4:** The Global Equilibrium Population (GEP, the average population of the nodes in the networks after equilibrium) loss after a shock is applied to a node with low, median or high centrality in the 10 node network. The increase in population in the shock node is deducted from this. The population loss in the rest of the network is larger when the shock is applied to a node with a higher centrality.

Node centrality	GEP loss in non-shock nodes
lowest	270
median	278
highest	285

## Discussion

### Climate variability, network topology and shocks

Our results show that resource redistribution (trade) increases the Node Equilibrium Population (NEP) and Global Equilibrium Population (GEP) of small-world networks when resources vary over time in an out of phase distribution. The GEP is higher when amplitude and frequency of variability are lower (LALF). Larger small-world networks are more efficient in redistributing variable resources, resulting in higher GEP. Larger networks also reduce inequality in NEP. Generally, more central nodes have higher NEP. Moreover, they are less susceptible to both climate-induced resource variability and shocks, because of their relative ease of access to resources compared with less central nodes. Shocks cause more damage to GEP when they are applied to more central nodes.

In line with previous research, our results show that trade in a small-world network can effectively deal with resource variability and increase carrying capacity [4; 8; 32]. However, our results add that larger small-world networks are more effective in mitigating climate variability and that more central nodes are less vulnerable to long-term resource variability and shocks.

Our results are in agreement with Suweis et al. (2015) [14] who also applied a Malthusian model and show that populations of importing nodes will grow to the carrying capacity, which is facilitated by exporting nodes. As soon as export ceases, resource availability reduces, resulting in population loss in the import-dependent nodes. However, for human populations, this is considered controversial. Lee et al. (2011) [33] argue that population growth is not restricted by resource availability but determined by other forces such as access to education, health care and social institutions. Nonetheless, a restriction on exports in times of shortage will reduce the carrying capacity of a trading network and has been shown to cause population loss, at least in pre-industrial times [34]. In our present-day society, shortages tend to lead to increased competition for food resources on regional and global markets and an associated price increase [10; 9]. Wealthy countries are robust and appear to be relatively well shielded against resource shocks [18]. Equally, there is generally a surplus of food on regional and global markets, meaning that populations are below regional and global carrying capacities, which provides a buffer against shortages. However, with continued growth in population and per-capita consumption as well as more extreme climate variability, this buffer may quickly disappear [35; 2]. Poorer and developing countries are more vulnerable in this case and at risk of failing to secure imports during periods of shortages on regional or global markets [36; 37; 18].

We also find that the dependency on fewer nodes makes a node more vulnerable to a shock. These results are in agreement with Marchand et al. (2016) [11] who found that both the intensification of trade and the distribution of food reserves influence the reaction to shocks. Marchand et al. (2016) [11] also show that food stocks can aid in reducing dependency on trade and vulnerability to shocks. We have not taken food reserves into account however, because in the past few decades the importance of food reserves in the resilience to shocks has decreased as stock sizes have decreased [14].

In the free-trade system we aimed to study, nodes will always reserve resources for export. This reserve was not counted toward the carrying capacity of the node. This is in disagreement with the model setup of Suweis et al. (2015) [14]. They also used the logistical growth equation of Verhulst [23], where the population logistically grows toward the carrying capacity. However, they added the exported resources to the carrying capacity, while these resources were not actually available for the country. This led to an increase in population in exporting countries, that therefore had to access the resources reserved for export to prevent famine. This decreased the amount of traded resources in the network, leading to population losses in import dependent countries. Applying this rule in our model led to a halt of trade in every scenario, because when the population in each node grew beyond its carrying capacity, all nodes decreased export and eventually stopped exporting. This always led to a situation with no trade. This is not realistic in the real world due to for example trade agreements and the fact that local production tends to be specialist and aimed at export [38; 39].

Our results suggest that increasing the network size is beneficial for all nodes in a food trade network and that increasing the number of links to a node increases the resilience of that node against both long-term climate variability and shocks. However, to increase resilience to shocks and climate variability for the whole network, an increase in network size (by adding trading partners) is necessary. This is because a larger network is more efficient in redistributing resources, effectively increasing the carrying capacity of the network. Much of the available data on trade is at country scale and therefore there is a tendency to think of trade as a process that occurs bilaterally among countries. However, although bilateral socio-economic relations between countries are important for facilitating trade, these bilateral trade patterns emerge as a result of the distribution of resources that move via finer scale infrastructural networks [6]. So, for cities in particular, it is important to expand the number of trading partners through building socio-economic relations at different scales and investing in trade infrastructure.

### Model setup

Several studies have recently examined the effects of shocks on real world food-trade networks through a combination of empirical and modelling approaches [e.g.] [18; 17; 16; 15]. These studies have focused on virtual water or staple food (wheat, soy, corn) trading networks and examined the effects of past shocks that have occurred.

Within these real-world networks there are many complex interdependencies, which make it challenging to isolate the impact of climate variability, network topology and shocks on the carrying capacity of the network [6]. For example, there are often power imbalances among trading partners owing to economic or political disparity that affect trade patterns following a shock. By analysing empirical data on the diffusion of trade shocks, Distefano et al. (2018) [18] found that poor countries suffer most from shocks in the network because of cross country income inequalities, and d’Amour et al. (2016) [16] found that these countries suffer most because they are import dependent on one or a few large exporters. There is also often a response of nodes in the network to shocks, with an increase in trade link diversity observed following a shock, which frequently leads to less vulnerability to future shocks [15]. Equally, the observed dynamics will be affected by other, unobserved dynamics in the network. For example, trade relations between countries may be established for product X, which will affect the trade in product Y among the same countries. However, observations of product Y may only be used in the study, which means that important factors driving the trade in product Y are neglected. These complexities within real-world data limit the possibility of isolating generalisable effects of certain variables such as climate variability, network topology and shocks on carrying capacity. To isolate and analyse these effects, a simple model was developed. In addition, this setup allowed us to assess the effects on the long-term dynamics that are relevant for population growth and multi-decadal climate variability, which is challenging due to the limited number of years for which trade data is available.

The simple modelling setup led to some limitations. For example, we modified the original gravity of trade equation by deleting demand as an input variable [22]. We made this modification because in a prototype version of the model that included demand within the trade equation, we observed that centrality led to a rich-get-richer positive feedback. The increased carrying capacity that arose from a central position, led to increased population and increased demand. Thus, where nodes have agency over other nodes, which is the case in real world networks, central nodes quickly out-compete more peripheral nodes. This suggests that the original gravity of trade equation is suitable for estimating flow between nodes on a short term, but not for determining carrying capacity in an evolving network.

Furthermore, the amplitudes of the resource variability scenarios were set to 5% and 50% of the average value for food production. The choice for amplitude values is less straightforward than for frequency, which is well known for different climatic phenomena. Even though the amplitude of these fluctuations might be known, the consequential effect on food harvests is dependent on a lot of variables, such as location, crop management, water management, disease etc. It is therefore difficult to determine the magnitude of the amplitude of food harvest that results from climate variability. We chose the values of 5% and 50% to reflect a low and high variability in resources.

## Conclusions

We studied the effect of climate variability, network topology and shocks on population growth in a model that simulates a resource redistribution in a small-world network. The results show that resource redistribution serves to increase carrying capacities and population size of the network when resource variability is out of phase between two parts of a network. This effect becomes stronger with increasing network size. The centrality of nodes (i.e. cities or countries) in the network correlates with population size, which is attributed to increased accessibility to resources through trade. These central nodes are also less vulnerable to resource variability and shocks, because they can access more resources as the paths to all the other nodes are relatively shorter. This means that centrality of a node improves the position of that node in the trade network, and therefore reduces their susceptibility to changes in the rest of the network. Nodes that depend on import from only a few other nodes, usually have a lower centrality. This enhances their risk of being affected by disturbances in the food supply of the global food trade network, especially when climate variability will increase in the near future.

Anthropogenic climate change will result in larger amplitude and higher frequency variations in food production. Especially the amplitude of variability has a major impact on carrying capacities. Trade in food networks is important because it potentially provides resilience to climate variability. However, the connectivity that trade imposes on the network needs to be managed to provide resilience to peripheral nodes and avoid the propagation of shocks through trade networks. At a node level, this can be achieved by increasing the number of trading partners, whilst at a global level carrying capacity can be increased by including more nodes in the network to increase network size.

## Supporting information

S1 Fig Flowchart of one iteration of the food trade model. This flowchart displays the calculations made for each node at each iteration of the model. The formulas that are used for calculation are presented in the methods section.
(TIF)

S2 Fig Cumulative distribution of NEP in the 10 node network under the resource variability scenarios. The cumulative fraction (y-axis) shows the fraction of nodes which have a lower NEP than X. Solid lines indicate in phase resource variability. Dashed lines indicate 180 degrees out of phase resource variability. Grey and purple areas represent the spread of 100 randomly created 10 node networks that serve as a sensitivity analysis.
(TIF)

S3 Fig Cumulative distribution of NEP in the 100 node network under the resource variability scenarios. The cumulative fraction (y-axis) shows the fraction of nodes which have a lower NEP than X. Solid lines indicate in phase resource variability. Dashed lines indicate 180 degrees out of phase resource variability. Grey and purple areas represent the spread of 100 randomly created 100 node networks that serve as a sensitivity analysis.
(TIF)

S1 File The food trade model and input variables. This ZIP file contains the model used to produce the results presented in this paper as well as the model to produce the sensitivity analysis.
(ZIP)
