# Supplementary figures and images for: The effects of network topology, climate variability and shocks on the evolution and resilience of a food trade network
Source: PLoS One. 2019 Mar 26;14(3):e0213378. doi: 10.1371/journal.pone.0213378 (PMC6435146; doi:10.1371/journal.pone.0213378)

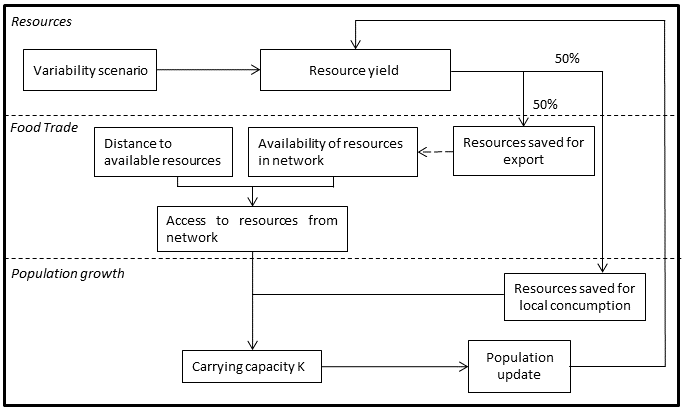

Supplement: S1 Fig — This flowchart displays the calculations made for each node at each iteration of the model. The formulas that are used for calculation are presented in the methods section. (TIF) [file pone.0213378.s001.tif]

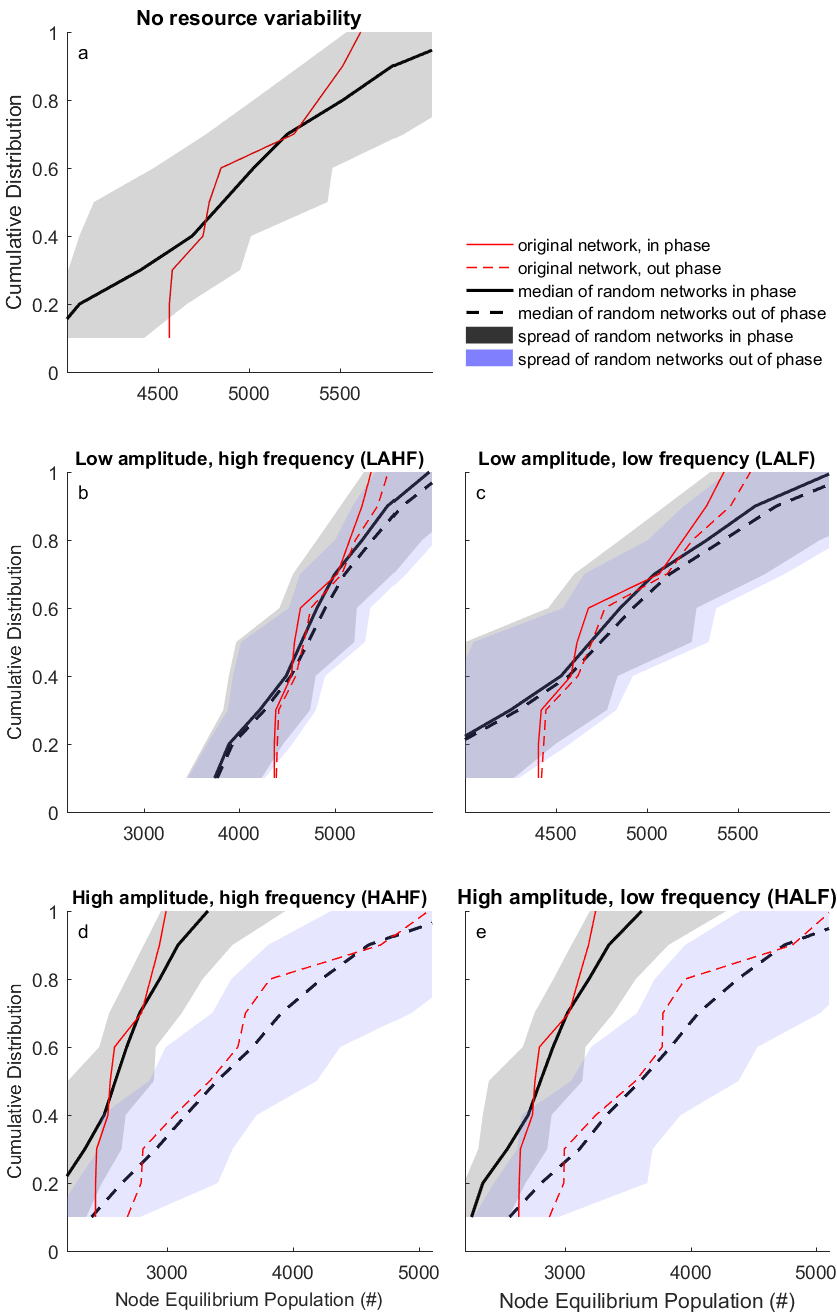

Supplement: S2 Fig — The cumulative fraction (y-axis) shows the fraction of nodes which have a lower NEP than X. Solid lines indicate in phase resource variability. Dashed lines indicate 180 degrees out of phase resource variability. Grey and purple areas represent the spread of 100 randomly created 10 node networks that serve as a sensitivity analysis. (TIF) [file pone.0213378.s002.tif]

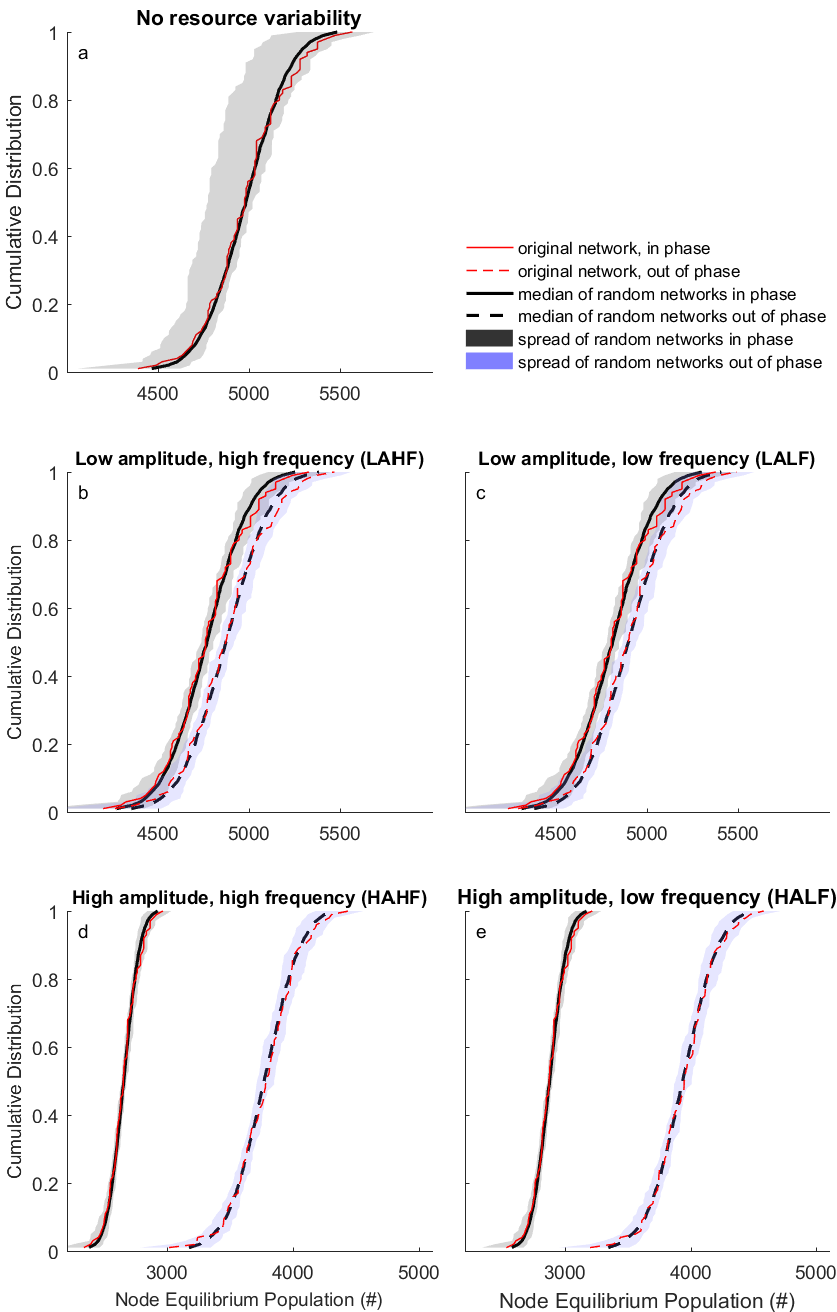

Supplement: S3 Fig — The cumulative fraction (y-axis) shows the fraction of nodes which have a lower NEP than X. Solid lines indicate in phase resource variability. Dashed lines indicate 180 degrees out of phase resource variability. Grey and purple areas represent the spread of 100 randomly created 100 node networks that serve as a sensitivity analysis. (TIF) [file pone.0213378.s003.tif]
